# Serum Metabolomic Profiles for Human Pancreatic Cancer Discrimination

**DOI:** 10.3390/ijms18040767

**Published:** 2017-04-04

**Authors:** Takao Itoi, Masahiro Sugimoto, Junko Umeda, Atsushi Sofuni, Takayoshi Tsuchiya, Shujiro Tsuji, Reina Tanaka, Ryosuke Tonozuka, Mitsuyoshi Honjo, Fuminori Moriyasu, Kazuhiko Kasuya, Yuichi Nagakawa, Yuta Abe, Kimihiro Takano, Shigeyuki Kawachi, Motohide Shimazu, Tomoyoshi Soga, Masaru Tomita, Makoto Sunamura

**Affiliations:** 1 Division of Gastroenterology and Hepatology, Tokyo Medical University, Shinjuku, Tokyo 160-0023, Japan; junko.umeda@gmail.com (J.U.); a-sofuni@amy.hi-ho.ne.jp (A.S.); tsuchiya623@mac.com (T.T.); g.shujiro@gmail.com (S.T.); onakasuicyatta@yahoo.co.jp (R.T.); tonozuka1978@gmail.com (R.T.); honjo3244@yahoo.co.jp (M.H.); moriyasu@tokyo-med.ac.jp (F.M.); 2 Institute for Advanced Biosciences, Keio University, Tsuruoka, Yamagata 997-0052, Japan; msugi@sfc.keio.ac.jp (M.S.); soga@sfc.keio.ac.jp (T.S.); mt@sfc.keio.ac.jp (M.T.); 3 Third Department of Surgery, Tokyo Medical University, Shinjuku, Tokyo 160-0023, Japan; kasuya-k@jcom.home.ne.jp (K.K.); naga@tokyo-med.ac.jp (Y.N.); 4 Fourth Department of Surgery, Tokyo Medical University Hachioji Medical Center, Hachioji, Tokyo 193-0998, Japan; abey3666@gmail.com (Y.A.); kiminoriman526@yahoo.co.jp (K.T.); skawachi@tokyo-med.ac.jp (S.K.); shimazu2401@yahoo.co.jp (M.S.); be7@xui.biglobe.ne.jp (M.S.)

**Keywords:** pancreatic cancer, biliary tract cancers, metabolomics, capillary electrophoresis mass spectrometry

## Abstract

This study evaluated the clinical use of serum metabolomics to discriminate malignant cancers including pancreatic cancer (PC) from malignant diseases, such as biliary tract cancer (BTC), intraductal papillary mucinous carcinoma (IPMC), and various benign pancreaticobiliary diseases. Capillary electrophoresis−mass spectrometry was used to analyze charged metabolites. We repeatedly analyzed serum samples (*n* = 41) of different storage durations to identify metabolites showing high quantitative reproducibility, and subsequently analyzed all samples (*n* = 140). Overall, 189 metabolites were quantified and 66 metabolites had a 20% coefficient of variation and, of these, 24 metabolites showed significant differences among control, benign, and malignant groups (*p* < 0.05; Steel–Dwass test). Four multiple logistic regression models (MLR) were developed and one MLR model clearly discriminated all disease patients from healthy controls with an area under receiver operating characteristic curve (AUC) of 0.970 (95% confidential interval (CI), 0.946–0.994, *p* < 0.0001). Another model to discriminate PC from BTC and IPMC yielded AUC = 0.831 (95% CI, 0.650–1.01, *p* = 0.0020) with higher accuracy compared with tumor markers including carcinoembryonic antigen (CEA), carbohydrate antigen 19-9 (CA19-9), pancreatic cancer-associated antigen (DUPAN2) and s-pancreas-1 antigen (SPAN1). Changes in metabolomic profiles might be used to screen for malignant cancers as well as to differentiate between PC and other malignant diseases.

## 1. Introduction

Pancreatic cancer (PC) has a poor prognosis. Its overall five-year survival rate is less than 5%, the lowest of all cancers [1]. The high mortality of patients with PC is attributable to its early dissemination, its lack of early, specific symptoms, and its late diagnosis [2]. Although recent diagnostic imaging technologies, such as computed tomography (CT), positron emission tomography-CT, magnetic resonance imaging and endoscopic ultrasonography (EUS) have contributed to improving the diagnosis of PC [3,4], many patients have locally advanced or metastatic disease at the time of diagnosis. Advances in chemotherapy including molecular targeted drugs and radiation therapy have ameliorated its prognosis [5,6], although surgery is still the only curative therapy for PC. Thus, the early detection of PC is of significant importance for improving its survival rate and prognosis.

Biliary tract cancer (BTC) is also a highly lethal malignant disease that arises from epithelial cells of the intrahepatic and extrahepatic bile ducts, which is increasing in incidence worldwide [7,8]. Although surgical operations for hepatic resection and liver transplantation are curative options, similar to PC, most patients are diagnosed at advanced stages. Thus, the development of low-invasive and frequent screening methods that enhance the opportunity to detect these cancers at early stages is urgent.

Blood testing is a simple, low invasive method for cancer screening. Currently, tumor markers including serum pancreatic enzymes and carbohydrate antigens such as CEA, CA19-9, DUPAN2 and SPAN1, are commonly used as a complement diagnosis [9,10,11]. However, many false negative cases occur even though these markers increase at the advanced stages of PC. Discrimination between PC and chronic pancreatitis (CP) is also difficult during mass forming pancreatitis and autoimmune pancreatitis despite the use of tissue diagnosis using endoscopic ultrasound-guided fine needle aspiration (EUS-FNA) [12,13]. These tumor markers do not help with decision-making for a diagnosis until surgery. Thus, the development of accurate early detection methods, such as molecular-based biomarkers, is highly desirable.

Metabolomics is an *omics* technology that enables the simultaneous quantification of hundreds of small molecules termed metabolites. Metabolite profiles, i.e., quantitative information of a metabolic pathway, directly reflect the phenotype of biological species and have been used for biomarker discovery [14,15]. Identification of metabolites to detect PC and BTC using a serum metabolomic profile has also been conducted intensively [16,17,18,19,20,21]. However, most of these comparisons included only healthy controls and PC patients, and in some cases with the addition of chronic pancreatitis. Evaluation of the specificity of discovered markers to other malignant cancers has not been reported. In addition, the reproducibility of quantified concentrations must be rigorously determined so this profiling technology can be used in clinical settings.

In this study, we conducted a comprehensive metabolomic analysis of serum to discriminate PC. Considering the potential clinical use of metabolomic data, we repeatedly measured metabolomic profiles of serum samples of different durations between collecting samples and measurement, to identify which metabolites had highly reproducible concentrations. Subsequently, we compared the profiles of these metabolites among patients with PC, BTC, and benign pancreaticobiliary diseases including chronic pancreatitis, and healthy controls. We developed and evaluated four mathematical models to discriminate these diseases from the other groups. 

## 2. Results

The characteristics of patients and healthy volunteers are summarized in Table 1. The study population was composed of 27 patients with pancreatic cancer (PC), 10 with BTC, two with intraductal papillary mucinous carcinoma (IPMC), six with CP, three with intraductal papillary mucinous adenoma (IPMA), 55 with benign pancreatobiliary diseases such as bile duct stone, ampullary adenoma or pancreatic pseudocyst and 46 healthy controls (C). PC, BTC, and IPMC were categorized into malignant disease (M), IPMA and other benign pancreatobiliary diseases were categorized into benign diseases (B).

**Figure 1:**
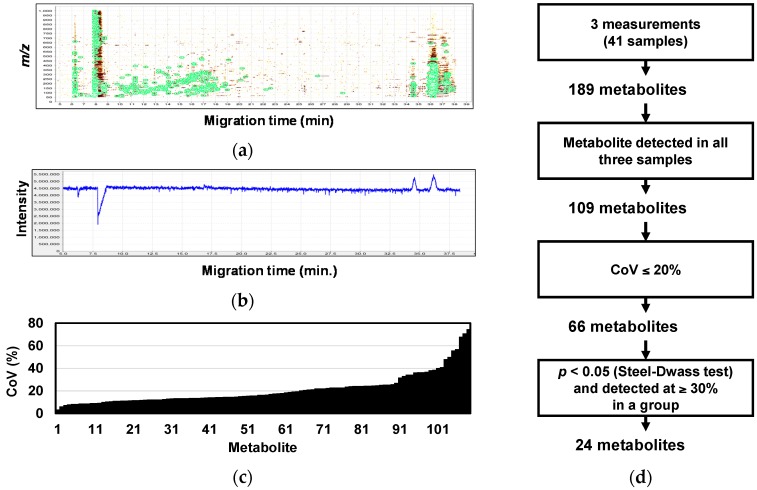
Typical capillary electrophoresis−mass spectrometry (CE-MS) data and data processing flow: (**a**) two-dimensional (2D) data of *m*/*z* and migration time showing detected peaks (green circles) and background signals (brown and yellow); (**b**) total ion electrophoresis; (**c**) distribution of coefficient of variation (CoV) for each metabolite; and (**d**) data processing flow for marker candidate selection.

Figure 1a,b shows typical capillary electrophoresis−mass spectrometry (CE-MS) data including two-dimensional data (migration time and *m*/*z*) and total ion electrophoresis (TIC) obtained from a patient with PC. Even though peaks were detected in two-dimensional data (green circles), TIC is almost constant except for the time when many salt ions were detected, since methanol peak in mass spectrum is exceptionally large compared to the other peaks.

Overall, 189 metabolites were successfully identified and quantified. To access the systematic bias derived from different storage durations, we measured randomly selected 41 samples, including C (*n* = 9), PC (*n* = 23), CP (*n* = 6), and IPMA (*n* = 3), three times each, and metabolites showing small coefficient of variation (CoV) were used for subsequent analyses. Of the profiled metabolites, 109 metabolites were consistently detected in three samples obtained from an individual. Overall, the profile showed a good CoV: 89 metabolites (81%, 89/109) had ≤25% CoV values (Figure 1c). Sixty-six metabolites had high stability (61%, 66/109) with ≤20% CoV. Of these, we selected 24 metabolites showing significant differences (*p* < 0.05; Steel–Dwass test) among C, M, and B, for subsequent analyses (Figure 1d and Table S1).

**Figure 2:**
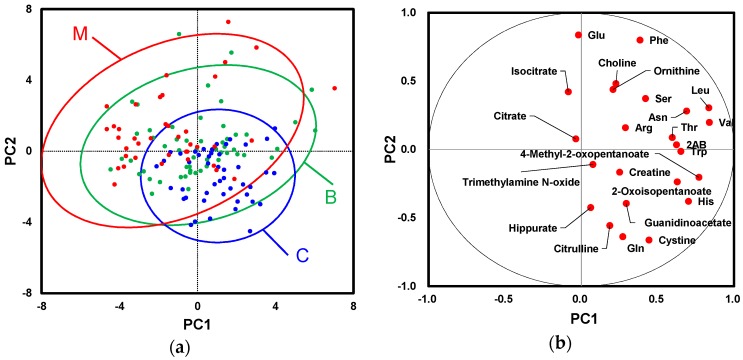
Principal component analysis (PCA) of serum metabolites: (**a**) score plots; and (**b**) loading plots. Contribution ratio of PC1 and PC2 were 20.7% and 14.1%, respectively. Ellipses on score plots indicated 95% confidential intervals of each group.

To determine changes in the overall patterns of metabolomic profiles, principal component analysis (PCA) was conducted. Each plot on score plots indicated the metabolomic profile of a serum sample (Figure 2a). Most of the plots of M (red) were observed in the upper left area (PC2 > 0 and PC1 < 0) while most C plots (blue) were observed in the lower right area (PC2 < 0 and PC1 > 0). Each plot of loading plots indicated each metabolite (Figure 2b). Except for isocitrate and citrate, all metabolites were distributed at right area (PC1 > 0) while these plots showed various PC2 values.

To identify the metabolite sets, i.e., combination of biomarkers, to discriminate between groups, we developed the following four multiple logistic regression (MLR) models.

- Model 1—to discriminate all diseases (M and B) from controls (C).
- Model 2—to discriminate malignant (M) from benign (B) disease.
- Model 3—to discriminate benign disease (B) from controls (C).
- Model 4—to discriminate pancreatic cancer (PC) from other malignant diseases (BTC and IPMC).

**Figure 3:**
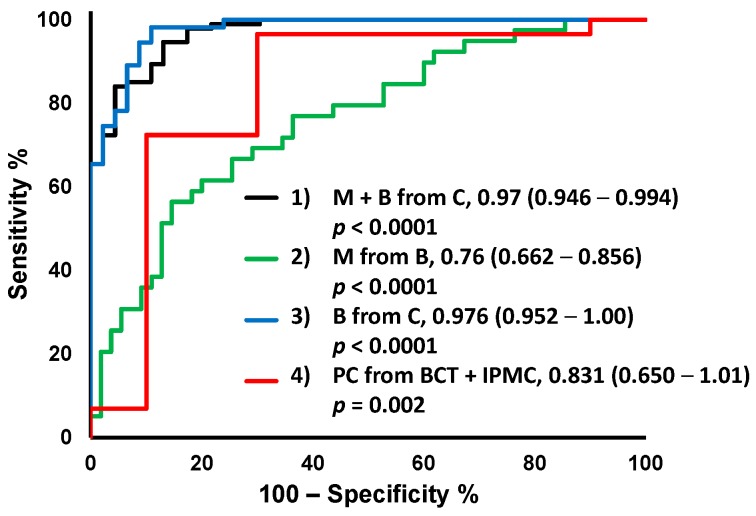
Receiver operating characteristic (ROC) curves of multiple logistic regression (MLR) models. The values are area under receiver operating characteristic curve (AUC), 95% confidence interval (CI) in parentheses, and *p*-value. C, healthy controls; M, malignant disease; B, benign disease; BTC, biliary tract cancer; IPMC, intraductal papillary mucinous carcinoma.

Receiver operating characteristic (ROC) curves of the models are shown in Figure 3. Model 1, which differentiated all disease groups from C, showed the highest value of area under ROC curve (AUC); 0.970 (95% confidential interval (CI); 0.946–0.994, *p* < 0.0001). Model 3 to discriminate B from C also showed a high AUC value; 0.976 (95% CI; 0.952–1.00, *p* < 0.0001). Models 2 and 4 showed lower AUC values compared to models 1 and 3. The metabolites used for each MLR model are listed in Table 2.

To evaluate the generalization ability of each MLR model, 200 10-fold cross validations (CV) were conducted. All models showed a small fluctuation of AUC values (Figure S1a). To eliminate optimistic prediction, we also conducted bootstrapping tests 200 times. Models 1 and 3 showed relatively small fluctuations of AUC values while Models 2 and 4 showed relatively large fluctuations in AUC values (Figure S1b).

**Figure 4:**
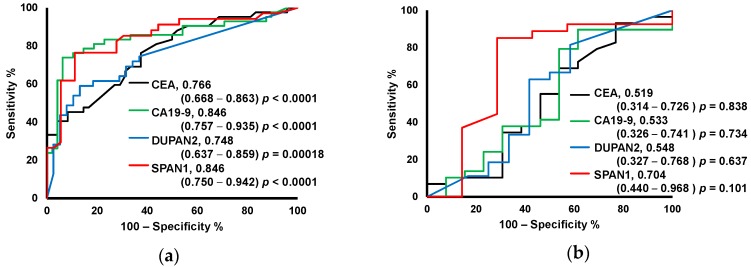
Prediction performance of MLR models. ROC curves to discriminate: (**a**) M (PC + BTC) (*n* = 42) from B (*n* = 52). The number of missing values of (PC and B) were (0, 4), (0, 4), (3, 14) and (8, 16) for carcinoembryonic antigen (CEA), carbohydrate antigen (CA19-9), pancreatic cancer-associated antigen (DUPAN2), and s-pancreas-1 antigen (SPAN1). (**b**) PC (*n* = 29) from BTC + IPMC (*n* = 13) by Model 1. The number of missing values of (PC and BTC + IPMC) were (0, 0), (0, 0), (2, 1) and (2, 6) for CEA, CA19-9, DUPAN2 and SPAN1, respectively. PC, pancreatic cancer; BTC, biliary tract cancer; ROC, receiver operating characteristic; IPMC: intraductal papillary mucinous carcinoma. The values are AUC, 95% CI in parentheses, and *p*-value.

Changes in tumor markers among patients groups were also observed. ROC curves discriminating M from B and PC from BTC + IPMC are shown in Figure 4a,b, respectively. The serum levels of CEA, CA19-9, DUPAN2, and SPAN2 were evaluated.

## 3. Discussion

Many studies to identify biomarkers for PC detection have been conducted using *omics* techniques. Serum metabolomics has an advantage for the screening of PC because the samples can be obtained low-invasively. Analyses of metabolomic profiles using partial least squares-discriminant analysis (PLS-DA)-based techniques, and a random forest model were reported to discriminate between two groups, e.g., PCs from controls [18,21,22,23,24,25,26,27,28]. These models can be used to determine the difference in overall profile between two groups. However, the problem of PLS-based methods uses all observed metabolites and discrimination model using fewer metabolites is preferable for considering the development of alternative assays. Therefore, in this study, we employed PCA to evaluate the overall profiles among multiple groups and developed MLR models to discriminate between groups using few candidate metabolite markers.

To screen for PC, a comparison of serum metabolites between PC and C was conducted [19]. Hopefully, the discrimination of PC and CP based on molecular biomarkers will contribute to the diagnosis of PC [29]. However, considering the complexity of pancreaticobiliary diseases, discrimination between PC and various benign diseases, such as autoimmune and gallstone pancreatitis, is also clinically important [21]. The current study undertook a rigorous evaluation, and compared M (PC and BTC) and variety of B diseases (CP, IPMA, bile duct stone (BDS), ampullary adenoma (AA), solid pseudopapillary neoplasm (SPN), adenomyomatosis (ADM), autoimmune pancreatitis (AIP), acute pancreatitis (AP), malfusion of pancreaticobiliary ducts (MPD), benign biliary stricture (BBS), pancreatic ductal stricture (PDS), and pancreatic pseudocyst (PPC)), and developed mathematical models based on various comparisons. PCA analyses showed a large difference in profiles between C and M, and B had an intermediate profile, which is in accord with the clinical phenotypes among C, B, and M.

All four metabolite-based MLR models showed discrimination ability at a significant level (Figure 3). Model 1 to discriminate M + B from C and Model 3 to discriminate B from C showed high AUC values (0.970 and 0.976, respectively). These models consist of a larger number of metabolites compared to the other models, which indicated the diversity of the metabolomic profiles in these groups and the difficulty of prediction using a single biomarker. Model 2 to discriminate M from B and Model 4 to discriminate PC from BTC + IPMC showed relatively low AUC values, even though they also reached statistically significant levels. The predicted probability of M was determined separately for PC, IPMC, and BTC (Figure S2a). The prediction accuracy was similar for each disease: AUC values were 0.975 (95% CI: 0.947–1.00, *p* < 0.0001), 0.975 (95% CI: 0.949–1.00, *p* < 0.0001), and 0.996 (95% CI: 0.984–1.00, *p* < 0.0001) for PC, IPMC, and BTC, respectively. There was also no stage-specific variation in the predicted probability of M for PC diseases (Figure S2b).

The sensitivity and specificity of CA19-9 ranged from 70% to 80% and 43% to 90%, respectively [11,30,31,32], for CEA from 42% to 45% and 75% to 78% [11,30], and for DUPAN2 they were 66% and 66% [11]. The current study evaluated the prediction ability of these tumor markers based on AUC values using identical cohort data so that the accuracy could be compared with metabolite-based predictive models. Tumor markers showed similar discrimination ability between M and B compared to metabolite-based MLR models. CEA and DUPAN2 had an AUC of 0.756 and 0.748, respectively. Model 2 (metabolites-based model to discriminate M from B) yielded an AUC of 0.760. CA19-9 and SPAN1 yielded an AUC of 0.846 for both cases. However, the tumor marker showed no predictive ability between PC and BTC + IPMC and all markers had low AUC values (0.519–0.704) that were not significant. Model 4 (metabolites-based model to discriminate PC from BTC + IPMC) had an AUC of 0.831 indicating the advantage of using multiple metabolite markers for the diagnosis of PC from other malignant tumors.

Malignant cancers were reported to have significantly different intermediate metabolites in metabolite energy metabolic pathways, e.g., amino acids, choline, and citrate, compared with normal tissues. This change was consistent with the nuclear magnetic resonance (NMR)-based serum metabolomics of BTC [20]. A comparison of serum metabolomics of PC and non-cancerous controls reported no significant difference in lactate, an end product of glycolysis that is usually activated in cancer cells, as well as a significant increase in choline and taurine, which are indicators of cancer and immune system responses, respectively, in PC patients [25]. Our data also consistently showed no significant difference in lactate and taurine (data not shown) and a significant increase in taurine in malignant cancers. A possible explanation for the inconsistency of these metabolites is the low reproducibility of lactate because of saturation and the low ionization of taurine in capillaries, which might confound quantification.

Model 1 included glutamic acid, histidine, glucamine, trimethylalanine *N*-oxide, and serine while Model 2 included creatine and guanidinoacetate. Change in these metabolites were consistent with other studies, such as a decrease in trimethylalanine *N*-oxide in the serum of PC patients [18]. Of note, we observed elevated histidine and phenylalanine in PC patients, whereas these metabolites were decreased in a previous study [29]. These differences should be further investigated using larger numbers and a variety of cohorts. There were several limitations in this study. The number of samples, especially PC and CP, were small and there was significant bias in several clinical variables, e.g., age and gender. In addition, advanced PC patients with stage III and IV were mostly included and, therefore, we have to confirm the results obtained in this study using larger cohorts including PC with early stage disease. Although many metabolites had a small CoV ≤ 25%, several metabolites had a large CoV including diethanolamine (71%) and citramalate (74%). Degradation of metabolites during sample storage, instability of standard compounds used for quantification, and the intensity of ionization of the metabolites might all account for the low stability. 

There are several limitations to be acknowledged. First, we used chloroform for metabolite extraction and organic solvents, such as lactate and glycerol, were educed from tube or filtration membrane [33]. Thus, these metabolites were not used for subsequent analyses. Second, although we identified corrected migration time and *m*/*z* values with matched compounds using single MS, the MS/MS-based fragment patterns of mass spectrum should be compared to increase the reliability of metabolite assignments. Thus, the current metabolite names were considered as tentative. Third, the quantification of metabolites was performed based on the calibration curves using various concentration of standard mixture, which indicated the correct quantified concentrations are available at only proper metabolite identification. In addition, we calculated concentration using single-point calibration while quantification with multiple calibration curve would be more accurate. In this study, we did not analyze peaks without matched standard library. The use of these data is our future work; however other quantification methods should be employed due to non-availability of the standard compounds. Lastly, we randomly selected 41 samples to evaluate the stability of quantified values of metabolites. However, the use of quality control samples using serum pool should save the consumption of samples.

In conclusion, our analyses revealed the concentrations of 24 metabolites were highly reproducible and their combinations showed highly discriminating ability for malignant cancers including PC, BTC, benign diseases, and healthy controls. One MLR model had high discrimination for M + B from C. Another MLR model successfully discriminated PC and other malignances (BTC and IPMC) with a higher accuracy than tumor markers. Taken together, serum metabolomic profiles might contribute to the screening of patients and the diagnosis of PC among malignant tumors.

## 4. Materials and Methods

### 4.1. Patient Selection and Serum Collection

Sample collection was conducted at Tokyo Medical University Hospital from January 2010 to December 2012. All patients had pancreaticobiliary cancers diagnosed histologically. All patients were recently diagnosed with primary disease and none had received any prior treatment in the form of chemotherapy, radiotherapy, surgery, or alternative therapy. No subjects had a history of prior malignancy. 

This study was approved by the ethics committee at Tokyo Medical University (approval no. 1560). Written informed consent was obtained from all patients and from volunteers who agreed to serve as sera donors. Our study was carried out in accordance with the Helsinki Declaration.

### 4.2. Sample Preparation

The collected blood was immediately centrifuged at 3000× *g* for 10 min at 4 °C, and the serum was transferred to a 1.2 mL polypropylene serum tube (SUMILON, Sumitomo Bakelite Co., LTD., Tokyo, Japan). Samples were stored at −80 °C until metabolomic analyses. Frozen samples were thawed and 40 µL aliquots of serum were mixed with 360 µL of methanol containing internal standards (20 µM each of methionine sulfone and camphor 10-sulfonic acid). The samples were vortex-mixed, and 400 µL of chloroform and 160 µL of Milli-Q water (Millipore, Billerica, MA, USA) were added with the mixer, followed by centrifugation at 10,000× *g* for 3 min at 4°C. MicroMixer E-36 (Taitec Co., Saitama, Japan) was used for vortex. The upper aqueous layer of each sample was filtered through a centrifugal filter tube with a 5-kDa cutoff (Millipore) at 9100× *g* for 2 hours at 4 °C to remove large molecules. The filtrates were concentrated by centrifugation for about 5 h at 40 °C using Acid-Resistant CentriVap Benchtop Centrifugal Vacuum Concentrator (Labconco Corp., Kansas, MO, USA) and resuspended in 40 µL of Milli-Q water containing other internal standards (3-aminopyrrolidine and trimesate) prior to CE-time-of-flight-MS analysis. The internal standards, 3-aminopyrrolidine and trimesate, were used for only correction migration time.

### 4.3. Measurement Conditions and Processing of Raw Data

The metabolite standards, CE-TOFMS instrumentation, and measurement conditions for cationic and anionic metabolites were described previously [34,35,36]. CE-TOFMS analysis was performed using an Agilent 7100 CE system (Agilent Technologies, Waldbronn, Germany), an Agilent 6224 LC/MS TOF system, an Agilent 1260 series isocratic HPLC pump, a G1603A Agilent CE-MS adapter kit, and a G1607A Agilent CE-ESI-MS sprayer kit (Agilent Technologies, Santa Clara, CA, USA). For system control and data acquisition, Agilent Chemstation software was used for CE and Agilent MassHunter software was used for TOFMS.

Cationic metabolites were separated on a fused-silica capillary column (50-µm inner diameter × 100-cm total length) filled with 1 M formic acid as the electrolyte. The sample solution was injected at 5 kPa for 3 s (approximately 3 nL), and a positive voltage of 30 kV was applied. The sheath liquid, methanol/water (50% *v*/*v*) containing 0.1 µM hexakis(2,2-difluorothoxy) phosphazen, was delivered at 10 µL/min. Anionic metabolites were separated using a commercially available COSMO(+) capillary column coated with a cationic polymer (Nacalai Tesque, Kyoto, Japan). Ammonium acetate solution (50 mM, pH 8.5) was used as the electrolyte. The sample solution was injected at 5 kPa for 30 s (approximately 30 nL) and a voltage of −30 kV was applied. The sheath liquid, ammonium acetate (5 mM) in methanol/water (50% *v*/*v*) containing 0.01 µM hexakis(2,2-difluorothoxy) phosphazen, was delivered at a rate of 10 µL/min. Results were automatically recalibrated relative to the masses of two reference standards in each mode. Cationic analysis used the ^13^C isotopic ion of a protonated methanol dimer (2MeOH + H)^+^, *m*/*z* 66.06306, and protonated hexakis(2,2-difluorothoxy) phosphazen (M + H)^+^, *m*/*z* 622.02896, whereas anionic analysis used the ^13^C isotopic ion of a deprotonated acetic acid dimer (2CH_3_COOH − H)^−^, *m*/*z* 120.03834, and hexakis(2,2-difluorothoxy) phosphazen + deprotonated acetic acid (M + CH_3_COOH − H)^−^, *m*/*z* 680.03554. Mass spectra were acquired at a rate of 1.5 cycles/sec from *m*/*z* 50 to 1000.

The processing of raw data, starting with converting vender-supplied raw data, noise-filtering, baseline-correction and peak-detection to integrate the peak area from sliced electropherograms, estimation of accurate *m*/*z*, alignment and peak matching across multiple datasets were conducted by our in-house software named MasterHands [37], which follows the typical data processing flow [38]. All compounds were identified by matching the *m*/*z* value and the corrected migration time to corresponding entries in a standard library. For all peak identifications, we confirmed the similarity of the isotope distributions between subjects and candidates. Concentrations were calculated using external standards based on relative area: the area divided by the area of the internal standards. Before the sample measurements, we measured mixture of our standard library including internal standards (methionine sulfone and camphor 10-sulfonic acid for cation and anion, respectively) and concentrations in serum samples were calculated by a single-point calibration method.

### 4.4. Stability Analysis of Metabolomic Profiles

To evaluate the stability of metabolomic profiles, randomly selected samples were measured three times at different days. CoV was calculated by the standard deviation divided by the mean for each metabolite. The mean CoV was calculated for the samples and used to select metabolites showing stable quantification values.

### 4.5. Data Analysis to Discriminate Disease Groups

Overall metabolomic profiles were determined by PCA. Comparison of the difference between two groups, e.g., C and PC, were assessed by the Mann–Whitney *U*-test. Multiple group comparisons (≥3) were assessed by the Steel–Dwass test for metabolomic data to detect a significant difference among the groups, and Kruskal–Wallis test and Dunn’s post-test for tumor markers. Nonparametric tests were used for both metabolomic data and tumor markers, considering non-Gaussian distributions. *p*-values calculated for metabolomic data were corrected by the false discovery rate (Benjamini and Hochberg methods) [39] yielding adjusted *p*-values, accommodating multiple independent tests.

MLR models were developed to discriminate between two groups. Forward and backward stepwise selection was used for models 1 to 3. *p* = 0.05 was used for the both thresholds; (1) to enter smallest *p*-value among the *p*-values for all covariates available to enter the model; and (2) to remove largest *p*-value among the *p*-values for all covariates currently selected for entry into the model. For model 4, forward stepwise selection (threshold; *p* = 0.05) was used because only a single marker was selected when forward and backward stepwise selection was used. Thus, totally 4 models were developed for 1 to 4.

To evaluate the versatility of the MLR models, we separated the data randomly into a training set (90%) and the remaining set (10%). We repeated this process up to 10 times for all data that were selected as validation data (10-fold CV). We repeated the 10-fold CV up to 200 times. To eliminate optimistic prediction, we also conducted bootstrapping analyses, by randomly selecting samples allowing redundant selections and yielding virtual cohorts. Overall, 200 virtual datasets with the same sample number were generated and the AUC values of the MLR model were calculated with already selected features.

JMP (Ver. 12.0.1, SAS Institute Inc., Cary, NC, USA), GraphPad Prism (Version 5.04, GraphPad Software, San Diego, CA, USA), XLSTAT (ver. 2014.1.09, Addinsoft, Paris, France), WEKA ver. 3.6.13 [40] and R package (Ver. 3.2.3) [41] were used for analyses.

## Figures and Tables

**Table 1:** Patient characteristics.

Group		*n*	Age ^1^	Gender ^2^	Stage
C	Healthy control	46	20–70 (31.5)	19/27	
M	Pancreatic cancer	27	51–83 (69.1)	13/14	(0/0/4/8/15) ^3^
	Biliary tract cancer	10	54–80 (70.0)	7/3	(1/1/2/1/5)
	Intraductal papillary mucinous carcinoma	2	70–80 (75.0)	1/1	(2/0/0/0/0) ^4^
B	Chronic pancreatitis	6	29–86 (63.9)	41/14	
	Intraductal papillary mucinous adenoma	3			
	Bile duct stone	17			
	Ampullary adenoma	15			
	Solid pseudopapillary neoplasm	1			
	Adenomyomatosis	2			
	Autoimmune pancreatitis	1			
	Acute pancreatitis	1			
	Malfusion of pancreaticobiliary ducts	1			
	Benign biliary stricture	4			
	Pancreatic ductal stricture	1			
	Pancreatic pseudocyst	3			

^1^ Min-max (average); ^2^ Male/female; ^3^ The numbers of patients were 16, 6, and 5 for head, body and tail of pancreas, respectively; ^4^ The number of patients were 0, 2, and 0 for head, body and tail of pancreas, respectively. C, healthy controls; M, malignant disease; B, benign disease; *n*, number.

**Table 2:** Parameters and odds ratios of each MLR model.

Model	Parameter	95% CI		Odds Ratio	95% CI		*p*-Value
**Model 1**							
(Intercept)	5.24	−0.379	11.5	-	-	-	0.078
Glu	0.0803	0.0495	0.124	1.08	1.05	1.13	<0.0001
His	−0.130	−0.216	−0.065	0.878	0.806	0.937	0.00050
Gln	0.0101	0.00267	0.0186	1.01	1.00	1.02	0.012
Trimethylamine *N*-oxide	0.0705	0.00519	0.133	1.07	1.01	1.14	0.022
Ser	−0.066	−0.114	−0.029	0.936	0.893	0.971	0.0019
**Model 2**							
(Intercept)	2.51	1.07	4.16	-	-	-	0.0013
Creatine	−0.0387	−0.0636	−0.0179	0.962	0.938	0.982	0.00080
Guanidinoacetate	−0.496	−0.944	−0.0973	0.609	0.389	0.907	0.020
**Model 3**							
(Intercept)	2.58	−6.23	11.5	-	-	-	0.56
Glu	0.0910	0.0526	0.149	1.10	1.05	1.16	0.00010
Cystine	−0.238	−0.471	−0.0658	0.788	0.624	0.936	0.018
Gln	0.0198	0.008	0.0370	1.02	1.01	1.04	0.0068
Arg	0.0531	0.0176	0.103	1.05	1.02	1.11	0.012
Trp	−0.106	−0.229	−0.0164	0.900	0.795	0.984	0.044
Ser	−0.0981	−0.177	−0.044	0.907	0.838	0.957	0.0029
**Model 4**							
(Intercept)	7.19	2.98	13.4	-	-	-	0.0051
Thr	−0.0334	−0.0686	−0.006	0.967	0.934	0.994	0.031
Isocitrate	−0.523	−1.16	−0.0265	0.593	0.312	0.974	0.061

MLR, multiple logistic regression; CI, confidence interval.

## Supplementary Materials

Supplementary materials can be found at www.mdpi.com/1422-0067/18/4/767/s1.

## Abbreviations

C	Control
M	Malignancy
B	Benign
HC	Healthy control
PC	Pancreatic cancer
BTC	Biliary tract cancer
IPMC	Intraductal papillary mucinous carcinoma
CP	Chronic pancreatitis
IPMA	Intraductal papillary mucinous adenoma
BDS	Bile duct stone
AA	Ampullary adenoma
SPN	Solid pseudopapillary neoplasm
ADM	Adenomyomatosis
AIP	Autoimmune pancreatitis
AP	Acute pancreatitis
MPD	Malfusion of pancreaticobiliary ducts
BBS	Benign biliary stricture
PDS	Pancreatic ductal stricture
PPC	Pancreatic pseudocyst
CoV	Coefficient of variation
PC	Principal component
PCA	Principal component analysis
MLR	Multiple logistic regression
CV	Cross validation
EUS-FNA	Endoscopic ultrasound-guided fine needle aspiration
TIC	Total ion electrophoresis
CE-MS	Capillary electrophoresis-mass spectrometry
ROC	Receiver operating characteristic
AUC	Area under receiver operating characteristic curve
