# Supplementary material for: Serum Metabolomic Profiles for Human Pancreatic Cancer Discrimination
Source: Int J Mol Sci. 2017 Apr 4;18(4):767. doi: 10.3390/ijms18040767 (PMC5412351; doi:10.3390/ijms18040767)
Supplement: Supplementary file 1 [file ijms-18-00767-s001.pdf]

Supplementary Materials: Serum Metabolomic Profiles for Human Pancreatic Cancer Discrimination

Takao Itoi, Masahiro Sugimoto, Junko Umeda, Atsushi Sofuni, Takayoshi Tsuchiya, Shujiro Tsuji, Reina Tanaka, Ryosuke Tonozuka, Mitsuyoshi Honjo, Fuminori Moriyasu, Kazuhiko Kasuya, Yuichi Nagakawa, Yuta Abe, Kimihiro Takano, Shigeyuki Kawachi, Motohide Shimazu, Tomoyoshi Soga, Masaru Tomita, Makoto Sunamura

Table S1. Metabolite concentrations (μM).

Metabolite	KEGG	C		M		B		Comparison [1]
		Mean	SD	Mean	SD	Mean	SD	
Glu	C00025	63.2	27.6	212	198	177	157	abde
His	C00135	76.38	12.64	54.14	10.77	62.40	13.30	abcde
Cystine	C00491	40.22	8.20	29.76	5.50	32.19	6.04	abde
4-Methyl-2-oxopentanoate	C00233	48.63	14.98	31.53	13.78	41.02	15.14	abcd
2-Oxoisopentanoate	C00141	12.24	3.46	8.99	2.80	10.53	3.38	abde
Thr	C00188	145.87	32.45	114.35	33.70	128.67	40.36	abde
Gln	C00064	631.16	88.64	491.69	218.71	550.08	173.51	ad
2AB	C02356	20.87	6.06	16.81	6.85	16.65	7.41	abe
Citrulline	C00327	33.79	12.34	24.87	9.06	29.14	10.21	ad
Creatine	C00300	58.02	69.75	32.80	18.25	51.96	28.58	acdf
Ornithine	C00077	69.36	22.92	88.17	30.80	85.15	21.29	abde
Arg	C00062	106.89	22.45	113.94	27.29	124.00	22.86	be
Choline	C00114	14.87	2.83	17.43	4.95	17.35	4.11	abe
Trimethylamine *N*-oxide	C01104	11.39	17.75	13.12	8.54	11.39	11.20	ad
Isocitrate	C00311	2.69	0.85	3.78	1.62	3.11	1.12	ad
Hippurate	C01586	3.96	3.89	2.59	3.47	2.20	3.27	be
Asn	C00152	52.20	9.08	46.01	10.70	46.98	10.08	abde
Phe	C00079	69.13	13.83	87.14	30.50	81.71	24.16	abd
Trp	C00078	57.70	11.63	48.50	15.14	54.06	13.64	ad
Ser	C00065	165.53	28.27	156.01	35.75	152.13	35.18	be
Leu	C00123	139.49	28.64	126.81	57.17	149.90	52.30	ac
Citrate	C00158	68.64	14.84	73.42	26.57	61.85	22.35	bc
Guanidinoacetate	C00581	2.65	0.59	2.20	1.17	2.76	1.18	c
Val	C00183	239.85	47.78	216.66	80.28	255.09	74.66	c

[1] Significant difference (adjusted $p < 0.05$) using the Steel–Dwass test between: (a) C and M; (b) C and B; (c) M and B; (d) C and M; (e) C and B; and (f) M and B. SD, standard deviation; C, healthy controls; M, malignant disease; B, benign disease.

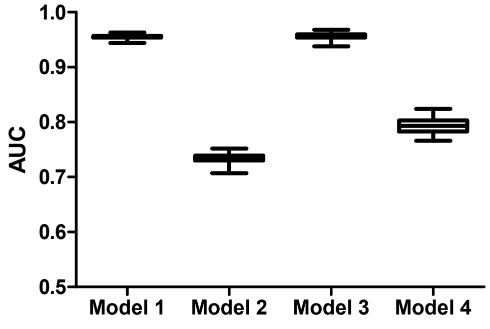

(**a**)

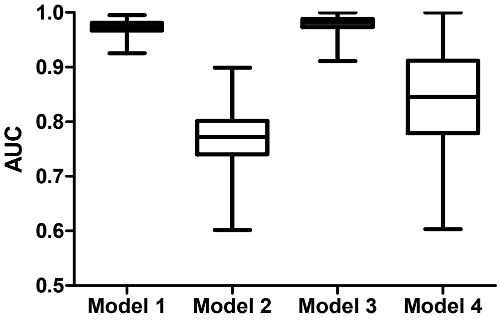

(**b**)

Figure S1. Distributions of AUC values of metabolite-based MLR models: (**a**) 200 repeats of 10-fold CV; and (**b**) 200 repeats of bootstrapping. AUC, area under the curve.

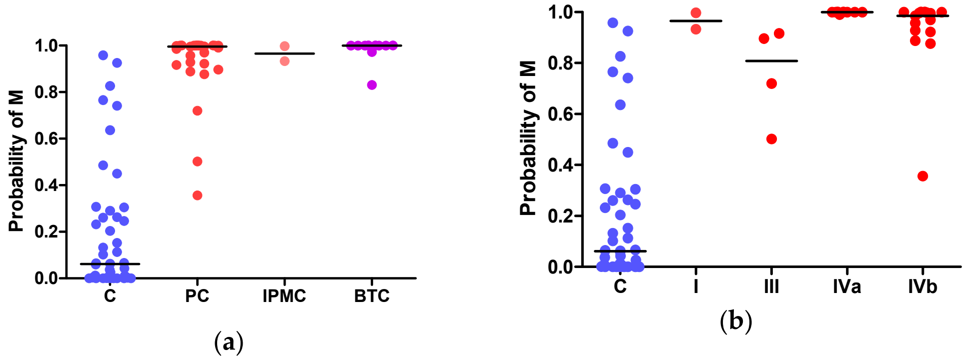

Figure S2. Distributions of probabilities of M predicted by model 1: (**a**) plots for C, PC, IPMC, and BCT; and (**b**) plots for C, and stages I, III, IVa, and IVb of PC. Stage II was not enrolled in this study. Lines indicated median value. C, healthy controls; M, malignant disease; PC, pancreatic cancer; BTC, biliary tract cancer; IPMC, intraductal papillary mucinous carcinoma.
