# Supplementary material for: Serum Metabolomic Profiles for Human Pancreatic Cancer Discrimination
Source: Int J Mol Sci. 2017 Apr 4;18(4):767. doi: 10.3390/ijms18040767 (PMC5412351; doi:10.3390/ijms18040767)
Supplement: Supplementary file 2 [file ijms-18-00767-s002.docx]

Supplementary Materials: Serum Metabolomic Profiles for Human Pancreatic Cancer Discrimination

Takao Itoi, Masahiro Sugimoto, Junko Umeda, Atsushi Sofuni, Takayoshi Tsuchiya,
Shujiro Tsuji, Reina Tanaka, Ryosuke Tonozuka, Mitsuyoshi Honjo, Fuminori Moriyasu, Kazuhiko Kasuya, Yuichi Nagakawa, Yuta Abe, Kimihiro Takano, Shigeyuki Kawachi, Motohide Shimazu, Tomoyoshi Soga, Masaru Tomita, Makoto Sunamura

**Table S1.** Metabolite concentrations (μM).

| **Metabolite** | **KEGG** | **C** | | **M** | | **B** | | **Comparison** ^1^ |
| --- | --- | --- | --- | --- | --- | --- | --- | --- |
|  |  | **Mean** | **SD** | **Mean** | **SD** | **Mean** | **SD** |  |
| Glu | C00025 | 63.2 | 27.6 | 212 | 198 | 177 | 157 | abde |
| His | [C00135](http://www.genome.jp/dbget-bin/www_bget?cpd:C00135) | 76.38 | 12.64 | 54.14 | 10.77 | 62.40 | 13.30 | abcde |
| Cystine | [C00491](http://www.genome.jp/dbget-bin/www_bget?cpd:C00491) | 40.22 | 8.20 | 29.76 | 5.50 | 32.19 | 6.04 | abde |
| 4-Methyl-2-oxopentanoate | [C00233](http://www.genome.jp/dbget-bin/www_bget?cpd:C00233) | 48.63 | 14.98 | 31.53 | 13.78 | 41.02 | 15.14 | abcd |
| 2-Oxoisopentanoate | [C00141](http://www.genome.jp/dbget-bin/www_bget?cpd:C00141) | 12.24 | 3.46 | 8.99 | 2.80 | 10.53 | 3.38 | abde |
| Thr | [C00188](http://www.genome.jp/dbget-bin/www_bget?cpd:C00188) | 145.87 | 32.45 | 114.35 | 33.70 | 128.67 | 40.36 | abde |
| Gln | [C00064](http://www.genome.jp/dbget-bin/www_bget?cpd:C00064) | 631.16 | 88.64 | 491.69 | 218.71 | 550.08 | 173.51 | ad |
| 2AB | [C02356](http://www.genome.jp/dbget-bin/www_bget?cpd:C02356) | 20.87 | 6.06 | 16.81 | 6.85 | 16.65 | 7.41 | abe |
| Citrulline | [C00327](http://www.genome.jp/dbget-bin/www_bget?cpd:C00327) | 33.79 | 12.34 | 24.87 | 9.06 | 29.14 | 10.21 | ad |
| Creatine | [C00300](http://www.genome.jp/dbget-bin/www_bget?cpd:C00300) | 58.02 | 69.75 | 32.80 | 18.25 | 51.96 | 28.58 | acdf |
| Ornithine | [C00077](http://www.genome.jp/dbget-bin/www_bget?cpd:C00077) | 69.36 | 22.92 | 88.17 | 30.80 | 85.15 | 21.29 | abde |
| Arg | [C00062](http://www.genome.jp/dbget-bin/www_bget?cpd:C00062) | 106.89 | 22.45 | 113.94 | 27.29 | 124.00 | 22.86 | be |
| Choline | [C00114](http://www.genome.jp/dbget-bin/www_bget?cpd:C00114) | 14.87 | 2.83 | 17.43 | 4.95 | 17.35 | 4.11 | abe |
| Trimethylamine *N*-oxide | [C01104](http://www.genome.jp/dbget-bin/www_bget?cpd:C01104) | 11.39 | 17.75 | 13.12 | 8.54 | 11.39 | 11.20 | ad |
| Isocitrate | [C00311](http://www.genome.jp/dbget-bin/www_bget?cpd:C00311) | 2.69 | 0.85 | 3.78 | 1.62 | 3.11 | 1.12 | ad |
| Hippurate | [C01586](http://www.genome.jp/dbget-bin/www_bget?cpd:C01586) | 3.96 | 3.89 | 2.59 | 3.47 | 2.20 | 3.27 | be |
| Asn | [C00152](http://www.genome.jp/dbget-bin/www_bget?cpd:C00152) | 52.20 | 9.08 | 46.01 | 10.70 | 46.98 | 10.08 | abde |
| Phe | [C00079](http://www.genome.jp/dbget-bin/www_bget?cpd:C00079) | 69.13 | 13.83 | 87.14 | 30.50 | 81.71 | 24.16 | abd |
| Trp | [C00078](http://www.genome.jp/dbget-bin/www_bget?cpd:C00078) | 57.70 | 11.63 | 48.50 | 15.14 | 54.06 | 13.64 | ad |
| Ser | [C00065](http://www.genome.jp/dbget-bin/www_bget?cpd:C00065) | 165.53 | 28.27 | 156.01 | 35.75 | 152.13 | 35.18 | be |
| Leu | [C00123](http://www.genome.jp/dbget-bin/www_bget?cpd:C00123) | 139.49 | 28.64 | 126.81 | 57.17 | 149.90 | 52.30 | ac |
| Citrate | [C00158](http://www.genome.jp/dbget-bin/www_bget?cpd:C00158) | 68.64 | 14.84 | 73.42 | 26.57 | 61.85 | 22.35 | bc |
| Guanidinoacetate | [C00581](http://www.genome.jp/dbget-bin/www_bget?cpd:C00581) | 2.65 | 0.59 | 2.20 | 1.17 | 2.76 | 1.18 | c |
| Val | [C00183](http://www.genome.jp/dbget-bin/www_bget?cpd:C00183) | 239.85 | 47.78 | 216.66 | 80.28 | 255.09 | 74.66 | c |

^1^ Significant difference (adjusted *p* < 0.05) using the Steel–Dwass test between: (a) C and M; (b) C and B; (c) M and B; (d) C and M; (e) C and B; and (f) M and B. SD, standard deviation; C, healthy controls; M, malignant disease; B, benign disease.

|   (**a**) |   (**b**) |
| --- | --- |

**Figure S1.** Distributions of AUC values of metabolite-based MLR models: (**a**) 200 repeats of 10-fold CV; and (**b**) 200 repeats of bootstrapping. AUC, area under the curve.

|   (**a**) |   (**b**) |
| --- | --- |

**Figure S2.** Distributions of probabilities of M predicted by model 1: (**a**) plots for C, PC, IPMC, and BCT; and (**b**) plots for C, and stages I, III, IVa, and IVb of PC. Stage II was not enrolled in this study. Lines indicated median value. C, healthy controls; M, malignant disease; PC, pancreatic cancer; BTC, biliary tract cancer; IPMC, intraductal papillary mucinous carcinoma.
